# High-speed colour-converting photodetector with all-inorganic CsPbBr_3_ perovskite nanocrystals for ultraviolet light communication

**DOI:** 10.1038/s41377-019-0204-4

**Published:** 2019-10-16

**Authors:** Chun Hong Kang, Ibrahim Dursun, Guangyu Liu, Lutfan Sinatra, Xiaobin Sun, Meiwei Kong, Jun Pan, Partha Maity, Ee-Ning Ooi, Tien Khee Ng, Omar F. Mohammed, Osman M. Bakr, Boon S. Ooi

**Affiliations:** 1 Photonics Laboratory, Division of Computer, Electrical, and Mathematical Sciences and Engineering, King Abdullah University of Science and Technology (KAUST), Thuwal, 23955-6900 Kingdom of Saudi Arabia; 2 Division of Physical Science and Engineering, King Abdullah University of Science and Technology (KAUST), Thuwal, 23955-6900 Kingdom of Saudi Arabia; 3 Quantum Solutions LLC, Thuwal, 23955-6900 Kingdom of Saudi Arabia

**Keywords:** Optoelectronic devices and components, Nanoparticles

## Abstract

Optical wireless communication (OWC) using the ultra-broad spectrum of the visible-to-ultraviolet (UV) wavelength region remains a vital field of research for mitigating the saturated bandwidth of radio-frequency (RF) communication. However, the lack of an efficient UV photodetection methodology hinders the development of UV-based communication. The key technological impediment is related to the low UV-photon absorption in existing silicon photodetectors, which offer low-cost and mature platforms. To address this technology gap, we report a hybrid Si-based photodetection scheme by incorporating CsPbBr_3_ perovskite nanocrystals (NCs) with a high photoluminescence quantum yield (PLQY) and a fast photoluminescence (PL) decay time as a UV-to-visible colour-converting layer for high-speed solar-blind UV communication. The facile formation of drop-cast CsPbBr_3_ perovskite NCs leads to a high PLQY of up to ~73% and strong absorption in the UV region. With the addition of the NC layer, a nearly threefold improvement in the responsivity and an increase of ~25% in the external quantum efficiency (EQE) of the solar-blind region compared to a commercial silicon-based photodetector were observed. Moreover, time-resolved photoluminescence measurements demonstrated a decay time of 4.5 ns under a 372-nm UV excitation source, thus elucidating the potential of this layer as a fast colour-converting layer. A high data rate of up to 34 Mbps in solar-blind communication was achieved using the hybrid CsPbBr_3_–silicon photodetection scheme in conjunction with a 278-nm UVC light-emitting diode (LED). These findings demonstrate the feasibility of an integrated high-speed photoreceiver design of a composition-tuneable perovskite-based phosphor and a low-cost silicon-based photodetector for UV communication.

## Perovskite-silicon receiver for UV wireless communication

A silicon-based receiver that incorporates perovskite nanocrystals efficiently detects ultraviolet signals, paving the way towards high-speed, high-bandwidth UV wireless communication. The photodetector (PD), developed by Boon S. Ooi of King Abdullah University of Science and Technology (KAUST) and colleagues in Saudi Arabia, is less bulky and cheaper to manufacture than currently available receivers. It builds on technologically advanced silicon-based PDs, which are compact and widely available, but respond best to higher wavelength green light. Incorporating cesium lead bromide (CsPbBr_3_) perovskite nanocrystals into a silicon-based PD facilitated efficient conversion of UV into green light. The team demonstrated that their receiver could be used in high-speed UV-based communication, paving the way for the use of perovskite-based materials in terrestrial and underwater UV-Internet systems.

## Introduction

Paving the way for fifth-generation (5G) wireless communication and beyond, which requires higher bandwidth and lower latency, optical wireless communication (OWC), including visible light communication (VLC) and ultraviolet (UV)-based communication, has attracted considerable attention. Compared to the existing regulated radio-frequency (RF) communication, OWC offers an unlicensed and secured bandwidth spanning UV to visible wavelengths of up to hundreds of THz to mitigate the congested bandwidth in the RF network^1^. For VLC, high data rates on the order of gigabits per second (Gbps) have been widely demonstrated using different modulation schemes in the line-of-sight (LOS) configuration^2–4^. However, this configuration by itself is inadequate for a complete communication system. Mimicking the indirect RF signal transmission pathway is pivotal in offering a robust solution for OWC. Fortunately, light in the UV wavelength region is highly scattered through both Rayleigh and Mie scattering, thus constituting the much-required non-line-of-sight (NLOS) communication pathway. This pathway will relieve the strict requirements on pointing, acquisition, and tracking in LOS communication^5,6^. Furthermore, UV-based communication is highly appealing compared to VLC owing to its low background solar radiation, particularly in the solar-blind UVC region (100–280 nm) due to the strong absorption by the ozone layer. Low noise-floor free-space communication in the UVC spectrum will also enable a wide variety of applications, such as missile detection^7^ and aircraft landing in low visibility conditions^8^. Moving forward, a reliable UV-based communication link, specifically in the UVC region, will create new frontiers for OWC systems, empowering the internet of things (IoT) and internet of underwater things (IoUT).

Despite the importance of a UV-based communication link, the limitations of transmitter and receiver technologies impede the current advancement. For the receiver end, high-performance photodiodes or multi-pixel detectors across the UV-to-visible region are crucial for various practical applications. In the OWC field, the bulky photomultiplier tube (PMT) is still being used owing to its large spectral range and high signal-to-noise (SNR) ratio^9^. However, it suffers from a high power consumption, a bulky form factor, and a high cost^10^. In contrast, compact and small-footprint group-III-nitride-based PDs^11^, e.g., AlGaN-^12^, AlN-^13^, and BN^14^-based PDs, suffer from costly materials and substrate development. The existence of defect states and crystal dislocations related to high dark current complicates the design process and delays the further deployment of these PDs in UV-based communication systems.

On the other hand, low-cost and technologically mature silicon (Si)-based PDs are widely and commercially available. Nevertheless, owing to the low penetration depth of high-energy UV photons in the silicon layer (e.g., <20 nm for the deep UV-to-UVA region)^15^, the responsivity of commercial Si-based PDs is less than 0.1 A/W for wavelengths below 400 nm. For practical communication in the UVC-to-UVA band, this low responsivity will degrade the SNR of the communication link.

In the following, recent efforts to circumvent the abovementioned issue are described for completeness, although it is noted that these techniques are not suitable for high-speed OWC systems. A conjugated polymer thin film was first used as a luminescent material for enhancing the UV responsivity of Si-based PDs by Levell et al.^16^. This method explores the use of luminescent thin films to absorb high-energy UV photons that are re-emitted in the visible-wavelength region, in which Si-based PDs exhibit a higher responsivity. Another down-converting luminescent material based on yttrium–vanadate–phosphate–borate:Eu (Y(V, PO_4_)_0.9_(BO_3_)_0.1_:Eu) integrated with Si-based PDs was also demonstrated in a previous study^17^; however, both luminescent materials are known to have a long decay time of up to the order of milliseconds^18,19^ and are thus not suitable for high-speed modulation. A nanopatterned luminescent solar concentrator for VLC was also investigated by Dong et al.^20^; however, the SuperYellow fluorescent emitter exhibits reduced absorption in the UV region, which limits its operation to only the visible-wavelength region. Zhang et al. also demonstrated the integration of MAPbBr_3_-based perovskite quantum dots with an electron multiplying charge coupled device-based image sensor^21^. Thus far, to the best of our knowledge, down-converting luminescent material for UV-based receivers, particularly those suitable for high-speed solar-blind UVC communication links, have not been investigated and demonstrated.

In contrast to other down-converting luminescent materials, all-inorganic lead halide perovskites (CsPbX_3_, where X = Cl, Br and I) have emerged as a new class of materials for optoelectronic applications owing to their facile solution-processable synthesis, controllable visible emission spectrum, high photoluminescence (PL) quantum yield (PLQY) and low optical gain threshold^22,23^. Moreover, recent approaches using a novel passivation technique^24^ and a Fabry-Perot microcavity^25^ on lead halide perovskite have also been shown to induce ultrastable amplified spontaneous emission and even lasing characteristics^22^. Such advancements are essential for realising high-performance optical devices, such as up-conversion lasers and high-resolution optical microscopes. CsPbBr_3_ nanocrystals (NCs) are among the most studied all-inorganic perovskites owing to their recently achieved highly air-stable characteristics compared with other organic halide perovskites, e.g., chloride- and iodide-based perovskite NCs^26^. In addition, CsPbBr_3_ NCs have a high absorption coefficient as well as a suitable bandgap for light detection and were recently demonstrated for use in a low-dose X-ray scintilator^27^. The abovementioned studies, however, do not shed light on the possibility of high-speed photodetection in the UVB–UVC band.

Herein, we present a novel receiver design for a UVC communication link using a hybrid CsPbBr_3_-silicon colour-converting photodetection scheme. The responsivity and external quantum efficiency (EQE) of Si-based PDs in the green spectrum region are significantly higher than those in the UV region. The high-PLQY drop-cast CsPbBr_3_ perovskite NC layer on a UV quartz substrate can effectively down-convert the incident UV light into the green wavelength region to realise an enhanced photodetection performance in the UV region. The measured small-signal modulation bandwidth of the hybrid PD confirmed the feasibility of high-frequency modulation. Correspondingly, a high-speed solar-blind UVC communication link was demonstrated using a 278-nm solar-blind UVC LED as a transmitter and the hybrid CsPbBr_3_–silicon colour converter as a receiver. Notably, the present study demonstrates the feasibility of using a high-PLQY and high-speed CsPbBr_3_ perovskite NC layer as a revolutionary colour-converting luminescent material in a mature silicon-foundry-based PD platform for high-sensitivity and high-speed UV-based communication. This game-changing and disruptive device technology is expected to eventually eliminate the need for the pursuit of a large bandgap semiconductor platform for high-speed UV photodetection.

## Results

**Fig. 1:**
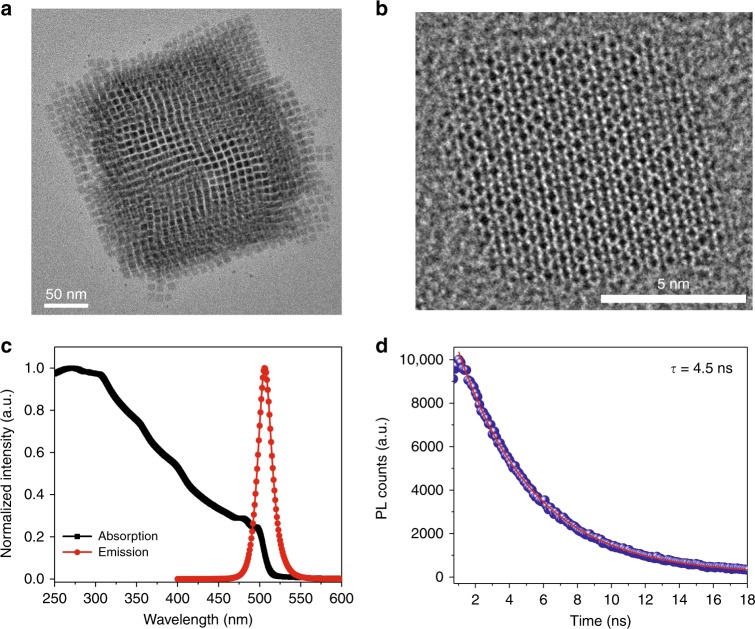
Structural and optical characterisation of CsPbBr_3_ perovskite NCs. **a** Transmission electron microscopy (TEM) image and **b** high-resolution TEM image of CsPbBr_3_ perovskite NCs. **c** Absorption and photoluminescence spectra, as well as **d** time-resolved PL decay trace monitored at 506 nm following 372 nm excitation for the drop-cast CsPbBr_3_ perovskite NCs on a UV quartz substrate

Figure 1a, b shows transmission electron microscopy (TEM) and high-resolution TEM (HR-TEM) images of the CsPbBr_3_ perovskite NCs used in the present study. The corresponding HR-TEM image shown in Fig. 1b reveals cubic NCs with an average size of approximately 6.39 ± 0.6 nm (see Supplementary Fig. S1 for a detailed image). Figure 1c shows the absorption and PL spectra of CsPbBr_3_ perovskite NCs. It is apparent that the CsPbBr_3_ perovskite NC layers have a sharp PL emission at approximately 506 nm, with a narrow full width at half maximum of 19 nm. At the same time, strong absorption in the UV region is also observed, which can be further validated by the PL excitation scan shown in Supplementary Fig. S2. In addition, the radiative recombination time between the photoexcited electron and hole of the CsPbBr_3_ perovskite NC layer was measured from the PL decay trace, which was monitored at 506 nm following 372 nm excitation (see Fig. 1d). The PL lifetime decay profile was collected at 506 nm. The decay curve can be fitted with a single exponential function with a lifetime of approximately 4.5 ± 0.1 ns, thus elucidating the potential of this layer as a high-speed luminescent material. In addition, we measured the PLQY of CsPbBr_3_ perovskite NCs dispersed in toluene and drop-cast onto a quartz substrate and found it to be near unity in the solution form, while it was reduced to ~73% in the thin-film form (see Supplementary Fig. S3). The high PLQY demonstrated in the case of the CsPbBr_3_ perovskite NC layer is significantly higher than that of the other down-converting materials for the Si-based receiver, for instance, aluminium tris-(8-hydroxyquinoline) (Alq3)^28^, *N,N’*-diphenyl-*N,N’*-bis-(3-methylphenyl)-1,1′-biphenyl-4,4′-diamine^28^, bis-(8-hydroxyquinaldine)-chlorogallium (Ga$q_2^{\prime}$Cl)^28^, fluorene copolymers^16^ and SuperYellow^20^. Compared to other organic-based luminescent materials, the improvement in terms of the PLQY is largely ascribed to the increased rate of recombination due to the reduced dimensionality (from a 3D bulk layer to 0D NCs), as well as to the unique synthesis method that results in reduced surface defects of NCs and surface passivation by oleic acid and oleylamine ligands. Using this method, the perovskite NCs are less susceptible to variations in the environment and thus yield a higher PLQY even in the form of thin films under ambient environments. Moreover, compared with commercialised CdSe-based NCs with a PLQY of ~30–52% and a PL decay time of a few tens of ns^29^, it is apparent that the PLQY achieved in CsPbBr_3_ perovskite NCs is significantly higher, concurrent with a faster PL decay time (<5 ns).

**Fig. 2:**
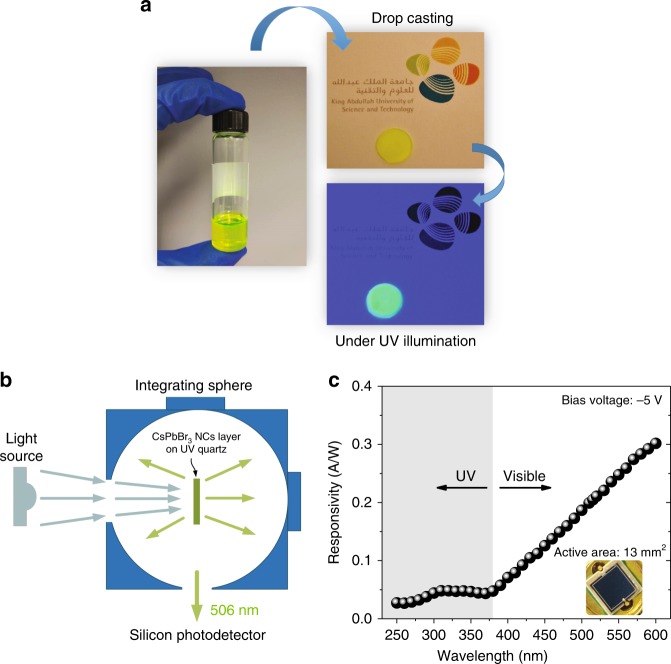
Novel photodetection scheme based on the hybrid CsPbBr_3_-Si platform. **a** Drop-cast CsPbBr_3_ perovskite NC layer on UV quartz under UV illumination. **b** Schematic illustration of the photoelectrical measurement setup of the Si-based PD with a CsPbBr_3_ perovskite NC layer on UV quartz inside the integrating sphere. **c** Measured responsivity spectrum of the bare Si-based PD

By taking advantage of the high-PLQY and short-PL lifetime, in the following sections, we explore the potential of CsPbBr_3_ NCs as a discrete component of a colour-converting luminescent material for high-speed Si-based PDs. Micrographs of the CsPbBr_3_ NC solution and drop-cast film on UV quartz when excited with a 278-nm UVC light source are shown in Fig. 2a. The transmittance of the UV quartz used in the present study can be found in Supplementary Fig. S4; >90% transmission can be observed from the UV-to visible-wavelength regions. For effective collection of the light re-emitted from the CsPbBr_3_ perovskite NC layer, the experimental setup shown in Fig. 2b was used to measure the photoelectrical performance of the Si-based PD with and without the addition of the luminescent material. The drop-cast CsPbBr_3_ perovskite NCs on the UV quartz were inserted into the integrating sphere. The inner surface of the integrating sphere was coated with polytetrafluoroethylene (PTFE), which is known to have >95% reflectivity from the deep-UV to infra-red wavelength regions. For proof of concept, a commercially available Si-based PIN junction PD (Thorlabs Inc., FDS100) with an active area of 13 mm^2^ was used for comparison. The uncoated borosilicate window was removed to expose the bare Si-based PIN structure. Figure 2c shows the measured responsivity of the commercial Si-based PD; it is evident that the responsivity drops significantly (<0.1 A/W) towards the UV region (<380 nm). This observation can be largely attributed to the low penetration depth, e.g., a few tens of nanometres, of UV light in the Si-based layer, as reported by Shi et al.^15^. To circumvent this issue, we aimed to down-shift the UV light absorbed by the high-PLQY CsPbBr_3_ NC layer into the green wavelength region, where Si-based PDs exhibit a higher responsivity of up to 0.2 A/W, as shown in Fig. 2c.

**Fig. 3:**
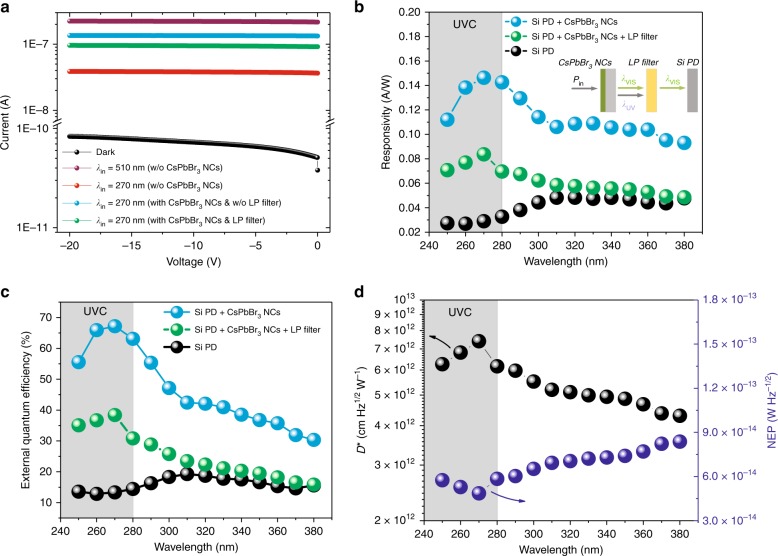
Performance of the hybrid CsPbBr_3_–Si photodetection scheme. **a** *I–V* curves of the Si-based PD with reverse bias from 0 to 20 V. The dark current is represented by the black line, while the red line represents the *I–V* curves with a 270-nm incident wavelength (*λ*_in_), and the purple line represents the *I–V* curves with a 510-nm incident wavelength. The enhanced photocurrent due to the down-conversion process of CsPbBr_3_ NCs measured with and without a 500-nm long-pass (LP) filter when excited by a 270-nm incident wavelength is represented by the green and cyan lines, respectively. **b** Comparison of the responsivity spectrum and **c** external quantum efficiency (EQE) for the bare Si-based PD and Si-based PD with the CsPbBr_3_ perovskite NC layer. The inset of **b** shows a schematic illustration of the photon conversion behaviour with an LP filter mounted between the CsPbBr_3_ layer in the integrating sphere and the Si-based PD (*P*_in_: incident light power, *λ*_VIS_: visible-wavelength photons, *λ*_UV_: UV-wavelength photons). **d** Specific detectivity (D*) and noise equivalent power (NEP) of the hybrid CsPbBr_3_–Si photodetection scheme in the UV wavelength region

Figure 3a shows the measured *I–V* characteristics of the Si-based PD under dark conditions (black line) as well as under 270-nm (red line) and 510-nm (purple line) excitation sources from 0 to 20 V (reverse bias). The incoming light intensity for both measurement wavelengths at 270 and 510 nm was calibrated to 8.5 µW/cm^2^ using neutral density (ND) filters. The photocurrent generated under excitation by the 270-nm light source is approximately an order of magnitude lower than that under excitation by the 510-nm light source. Notably, with the addition of the CsPbBr_3_ perovskite NC layer on the highly UV-transparent quartz substrate in the integrating sphere, the down-conversion approach allows higher absorption by the Si-based PD even under excitation by the 270-nm light source, thus generating a higher photocurrent (see the cyan line in Fig. 3a), distinctly closer to that under excitation by the 510-nm source. However, when the CsPbBr_3_ layer is under illumination, a fraction of absorbed photons is re-emitted at a longer wavelength depending on the quantum yield of the enhancement layer, while the remaining fraction of unabsorbed or scattered photons can escape into the Si-based PD without any photon conversion process^16^. These photons will result in additional carriers photogenerated from the two distinct wavelengths (e.g., 510 and 270 nm) in the Si-based PD. To evaluate the actual photogenerated carriers of the proposed colour-converting scheme based on the CsPbBr_3_ NC layer, we also measured the resultant *I–V* characteristics with a 500-nm long-pass (LP) filter (Thorlabs, FELH0500) mounted between the integrating sphere and the Si-based PD. By using the LP filter to prevent the unabsorbed UV-wavelength photons from being detected by the Si-based PD, as shown in Fig. 3a (see the green line), the photogenerated carriers remain higher than those under the 270-nm illumination without the CsPbBr_3_ NC layer. Using the integrating sphere, we also measured the responsivity of the Si-based PD with and without the addition of the CsPbBr_3_ perovskite NC layer in the UV wavelength region. The responsivity (*R*) is a key figure of merit for PDs, and it can be calculated on the basis of the generated photocurrent (*I*_photocurrent_) and incident light power (*P*_incident_) as follows:

1 $$R = \frac{{I_{\rm{photocurrent}}}}{{P_{\rm{incident}}}}$$

Figure 3b shows the responsivity spectrum of the bare Si PD and the PD with the insertion of the drop-cast CsPbBr_3_ perovskite NCs in the integrating sphere over the entire UVC-to-UVA region (250–380 nm). The measurement was taken at 10-nm intervals, and the incident light intensity was calibrated to approximately 8.5 µW/cm^2^. As shown in Fig. 3b, the overall responsivity value based on the CsPbBr_3_ NC layer without the LP filter is demonstrated to be well above 0.1 A/W; however, this high responsivity value is subject to two combined factors of re-emitted visible photons (*λ*_VIS_) and unabsorbed UV photons (*λ*_UV_) that contribute to additional photogenerated carriers. By using the LP filter, we measured the actual value of the responsivity based on the CsPbBr_3_ NC colour-converting scheme, i.e., *λ*_UV_ to *λ*_VIS_. It is apparent that the responsivity remains nearly threefold higher (i.e., from 29 to 84 mA/W at 270 nm) than that of the bare Si-based PD, particularly in the UVC wavelength region, where the Si-based PD is known to exhibit low responsivity. This improvement can be attributed to the high PLQY and high UVC absorption of the CsPbBr_3_ NC layer. The responsivity decreases towards longer wavelengths due to the reduced light absorption at the near band edge of the CsPbBr_3_ NC layer, which is beyond the intended UV operating wavelength. Nevertheless, our work is comparable to that of Levell et al.^16^, who used an organic-based luminescent material. The organic-based layer exhibits a reduced photoresponse between 250 and 300 nm due to the dip originating from the absorption spectrum. Comparatively, as shown previously in Fig. 1c, the absorption spectrum of the CsPbBr_3_ perovskite NC layer increases gradually towards the shorter wavelength region, and thus, the photoresponse remains relatively stable even in the deep UV wavelength region. Moreover, the organic-based layer is also known to have a long PL decay time, which may limit its practical application in high-speed UV-based communication. The enhanced responsivity could significantly improve the sensitivity and SNR ratio of the PDs, particularly in detecting a low-intensity UVC light source.

Furthermore, we calculated the photon conversion efficiency, which is also known as the EQE, of the Si-based PD with and without the addition of the CsPbBr_3_ perovskite NC layer in the integrating sphere. The EQE is the ratio of the electron–hole pairs generated by the photodiode to the incident photons

2 $${\rm{EQE}} = \frac{{R_\lambda }}{{\lambda _{\rm{incident}}}} \times \frac{{hc}}{e}$$

where *R*_*λ*_ is the responsivity in A/W, *λ*_incident_ is the excitation wavelength in nanometres, *h* is Planck’s constant, *c* is the speed of light in vacuum, and *e* is the elementary charge. Based on the measured responsivity, the calculated EQE for the bare Si-based PD and PD with the inclusion of the CsPbBr_3_ perovskite NC layer in the integrating sphere is shown in Fig. 3c. By using the CsPbBr_3_ perovskite NCs, without an LP filter, a high EQE of up to 67% is observed at 270 nm, ascribed to the additional photogenerated carriers in the Si-based PD arising from the unabsorbed UV photons^16^. Thus, to evaluate the EQE based on the proposed colour-converting scheme, an LP filter must be mounted between the integrating sphere and the Si-based PD. With this setup, the EQE based on the CsPbBr_3_ colour-converting layer is measured to be approximately 25% higher than that of the bare Si-based PD in the UVC region. The significantly improved EQE in the UVC wavelength region is attributed to the enhanced photon absorption and increase in the photogenerated carriers after the photon conversion process. It is also worth noting that the carrier multiplication process is not evident because the EQE of the PD in the UV wavelength region remains lower than that in the green wavelength region, i.e., ~46.41% at 510 nm. The EQE peak observed at approximately 270 nm corresponds well with the peak position of the absorption spectrum, as shown previously in Fig. 1c, where it exhibits higher photon absorption than that beyond 300 nm. This phenomenon results in a higher number of photons being converted to a longer wavelength, where the Si-based PD exhibits a higher responsivity. The absorption coefficient at 270 nm was estimated to be 1.16 × 10^3^ cm^−1^, which is comparatively higher than that in the 350 nm band of approximately 0.89 × 10^3^ cm^−^^1^. The estimated absorption coefficient across 250 to 600 nm is included in Supplementary Fig. S5. As demonstrated by Maity et al.^30,31^, another important parameter to evaluate the performance of a PD, i.e., the specific detectivity (D*) was also calculated based on Eq. (3) as shown below

3 $$D \! ^{\ast} = \frac{{A^{\frac{1}{2}}}}{{\rm{NEP}}} = \frac{{(R_\lambda )(A^{\frac{1}{2}})}}{{(2eI_d)^{\frac{1}{2}}}}$$

where *A* is the device area in cm^2^, NEP is the noise equivalent power (NEP) in W Hz^−^^1/2^, *e* is the elementary charge of 1.602 × 10^−19^ coulombs, *I*_*d*_ is the dark current in amperes and *R*_*λ*_ is the responsivity in A/W^30–32^. The D* of the hybrid CsPbBr_3_–Si photodetection scheme, as shown in Fig. 3d, is determined to be 7.4 × 10^−^^12^ cm Hz^1/2^ W^−1^ at 270 nm, which is higher than that of the bare Si photodetection scheme, with a value of 2.5 × 10^−12^ cm Hz^1/2^ W^−^^1^ at the same measurement wavelength. The comparison of the specific detectivity between bare Si and the hybrid CsPbBr_3_–Si photodetection scheme is shown in Supplementary Fig. S6. In addition, the noise-related performance of the proposed scheme was also evaluated based on the NEP. NEP is defined as the amount of input optical power that generates an output photocurrent equal to the noise current to yield an SNR of 1. As calculated based on Eq. (3), in the UVC region, the NEP for the bare Si-based photodiode was calculated to be as high as 1.49 × 10^−13^ and 1.40 × 10^−13^ W Hz^−1/2^ for 250 and 270 nm, respectively. By using the proposed colour-converting scheme with CsPbBr_3_ perovskite NCs, as shown in Fig. 3d, the NEP is reduced by more than a half-order of magnitude to 5.75 × 10^−14^ and 4.86 × 10^−^^14^ W Hz^−^^1/2^ for the same measurement wavelengths at 250 and 270 nm. The comparison of the NEP between bare Si and the hybrid CsPbBr_3_–Si photodetection scheme is shown in Supplementary Fig. S7. By using the proposed colour-converting scheme, the lower NEP compared to that of the bare Si-based PD contributes to the lower noise floor and enhanced detectivity, particularly in the UVC wavelength region. A comparison of the device performance for various commercial and modified Si-based PDs is also summarised in Supplementary Table S1. In our work, based on the high PLQY of the CsPbBr_3_ perovskite NCs, the incremental responsivity and EQE were compared to those of a commercial Si-based PD. By using the perovskite phosphor, a similar method could be employed for all the other unmodified and modified Si-based PDs to realise improved performance, thus revolutionising the UV-based photodetection methodology using low-cost Si-based PDs. To further investigate the photostability of the CsPbBr_3_ perovskite NC layer for the UV-based communication link, we performed a 24-h PL stress test in an ambient environment under intense UVC illumination, as shown in Supplementary Fig. S8. The gradual increase in the PL intensity in the first 3 h could be attributed to the evaporation of reduced solvents and the formation of additional emissive centres linked to the dynamics of CsPbBr_3_ perovskite NCs^33,34^. In the subsequent 12 h, the PL intensity remained stable under ambient conditions. The photostability exceeds that of other untreated CsPbBr_3_ perovskite NCs, which degrade within a few hours^35–38^. The reduction of the PL intensity after 16 h of intense continuous irradiation under a focused UVC light source is most likely due to the thermal degradation and photooxidation of lead atoms^33,39,40^. Further improvement of the photostability of the CsPbBr_3_ film is undoubtedly possible by employing a core/shell structure^38^, surface passivating ligands^24,33^ and encapsulation^35^, as demonstrated in other prior works. For instance, Sun et al. demonstrated highly stable CsPbBr_3_ perovskite NCs under intense blue-LED light illumination of 175 mW/cm^2^ based on the sequential surface absorption method^37^. The coating of strongly hydrophobic silicone resin on perovskite-based NC thin films has also been demonstrated to improve the photostability and water resistance properties, as demonstrated by Hai et al.^36^. These facile and general strategies could open up a new avenue for highly air- and moisture-stable perovskite NCs.

**Fig. 4:**
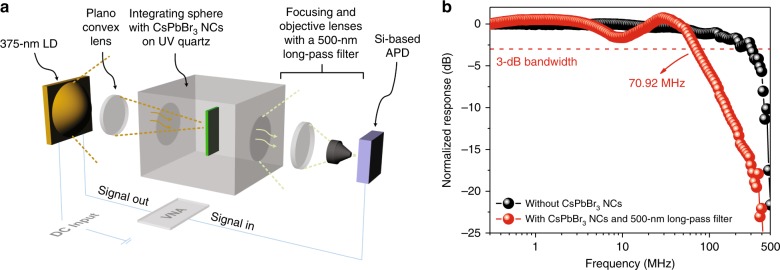
Modulation bandwidth measurement using CsPbBr_3_ perovskite NCs. **a** Schematic diagram of the small-signal frequency response measurement setup using a 375-nm UV laser diode (LD) and a Si-based avalanche photodiode (APD) to obtain the results in (**b**). **b** The normalised frequency response of the UV LD and APD without CsPbBr_3_ perovskite NCs and a 500-nm long-pass filter is shown by the black line. The red line shows the frequency response of CsPbBr_3_ perovskite NCs measured using the UV LD and APD with the UV light filtered by a 500-nm long-pass filter

To investigate the potential application of the CsPbBr_3_ perovskite NC layer to the UV-based communication link, we measured the small-signal modulation bandwidth of the CsPbBr_3_ perovskite NCs using the setup shown in Fig. 4a. To eliminate the bandwidth constraints from the emitter and other parts of the receiver, we used a 70-mW 375-nm UV laser diode (LD) with a high-modulation bandwidth of up to a few GHz and a Si-based avalanche PD (APD) with a device area of 0.2 mm and a tuneable gain of up to 5 × 10^5^ V/W. The UV light was guided through a plano-convex lens and focused onto the CsPbBr_3_ perovskite NC layer inside the integrating sphere. Another series of plano-convex lenses and an objective lens was set up at the output port to collect the photons re-emitted from the CsPbBr_3_ perovskite NC layer. Before entering the APD, the UV light was filtered using a 500-nm LP filter. By sweeping the sinusoidal AC modulation signal from 300 kHz to 3 GHz, as shown in Fig. 4b, the 3-dB bandwidth of the system without CsPbBr_3_ perovskite NCs is determined to be approximately 380 MHz. With the addition of the CsPbBr_3_ perovskite NC layer and 500-nm LP filter, a 3-dB bandwidth of approximately 70.92 MHz is obtained. The gradual dip at 10 MHz arises from the overall frequency response of the system when the CsPbBr_3_ perovskite NCs are inserted into the integrating sphere. The origin of this phenomenon has not been explicitly identified, although it is commonly observed when additional materials, optics or optical components are inserted into the communication channel. Such a gradual dip is within the −3 dB bandwidth, and this can be corrected through power pre-equalisation or post-equalisation in the eventual system implementation using electronic hardware or software processing. Apart from the enhanced responsivity relative to the Si-based PD, we posit that the demonstrated bandwidth has great potential for integration with a high-speed Si-based PD targeted at the UVC communication link. By taking advantage of the short carrier recombination lifetime (~ns), the demonstrated modulation bandwidth under UV excitation is significantly higher than that in the case of YAM:Eu^3+^-based (~ms)^41^, Sr_5_(PO_4_)_3−*x*_(BO_3_)_*x*_Cl:0.04Eu^2+^-based (~µs)^19^, and Ca_8_MgLu_1−*x*_(PO_4_)_7_:*x*Tb^3+^-based (~ms) phosphors^18^. The high-modulation bandwidth demonstrated in CsPbBr_3_ perovskite NCs also surpasses those of other colloidal semiconductor NC-based PDs, e.g., CdSe quantum dots (~50 kHz)^42^. Moreover, although some of the published studies have demonstrated a high modulation bandwidth of up to hundreds of megahertz^20,43,44^, reduced absorption and a low PLQY in the UV region were also observed, which restricts the application of these materials in the UV communication field.

**Fig. 5:**
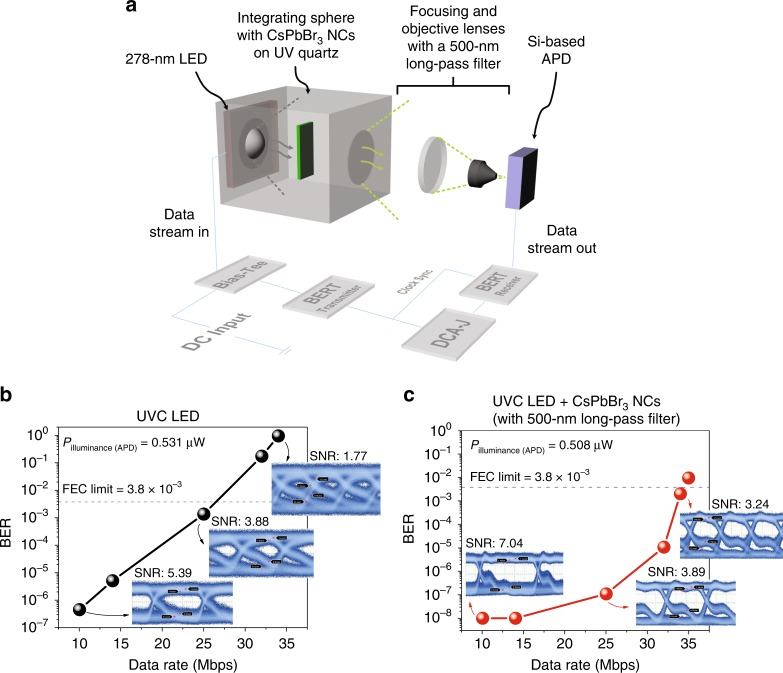
Data transmission measurement using CsPbBr_3_ perovskite NCs. **a** Schematic diagram of the data transmission measurement setup with an on-off keying (OOK) modulation scheme using a solar-blind 278-nm UVC LED and a Si-based APD. **b** Bit-error rate (BER) of data transmission at different data rates without a CsPbBr_3_ perovskite NC layer and a 500-nm long-pass filter. For comparison, an optical density (OD) filter was added to ensure that the optical power illuminating the APD is the same as that in the case of CsPbBr_3_ perovskite NCs and is below the saturation limit of the APD. The insets show the corresponding eye diagrams. **c** BER of data transmission at different data rates with the colour-converting CsPbBr_3_ perovskite NC layer and UV light filtered by a 500-nm long-pass filter. The insets show the corresponding eye diagrams

We further demonstrated the potential of the CsPbBr_3_ perovskite NC layer as a colour-converting luminescent material for Si-based PDs in the solar-blind UVC communication link by using the on–off keying (OOK) modulation scheme. In the OOK modulation, a pseudorandom binary sequence 2^10^–1 data format was sent to optically modulate the transmitter, where the 1 or 0 s of the digital data were represented by the presence or absence of the carrier wave, respectively. As shown in Fig. 5a, we used a 278-nm UVC LED to excite the CsPbBr_3_ perovskite NC layer in the integrating sphere, while the green luminescence was passed through a series of plano-convex lenses, an objective lens, and a 500-nm LP filter before entering the Si-based APD. A DC bias of 6 V and an AC modulated peak-to-peak voltage of 2 V_p–p_ were supplied to the LED. At 6-V DC bias, the emitted light output power was measured to be approximately 0.8 mW. The corresponding *L–I–V* curves and light emission spectrum of the 278-nm LED can be found in Supplementary Fig. S9. At both the transmitter and receiver ends, the devices were connected to a bit-error-rate (BER) tester. The distance between the UVC LED and the CsPbBr_3_ perovskite NC layer was 3.5 cm, while the distance from the CsPbBr_3_ perovskite NC layer to the Si-based APD was approximately 20 cm. For comparison, we also measured the BER achieved at different data rates in the case of the UV LED only without the CsPbBr_3_ perovskite NC layer and 500-nm LP filter, as shown in Fig. 5b. We used an optical density (OD) filter of 1.6 to reduce the optical power illuminating the APD, where the measured illuminated power before the APD was approximately 0.531 µW, well below the saturation limit of the APD. Prior to this, the system bandwidth was also measured, as shown in Fig. S10, where the 3-dB bandwidth was limited to 11.13 MHz owing to the limitations at the transmitter end. In the case of the UVC LED only, the highest achievable data rate was recorded as 25 Mbps, with a measured BER of 1.4 × 10^−3^, below the forward error correction (FEC) limit of 3.8 × 10^−3^. The inset of Fig. 5b shows the corresponding eye diagram with an SNR ratio of approximately 3.88 and a near-closed eye diagram for data rates above the FEC limit. With the colour-converting CsPbBr_3_ perovskite NC layer, higher data rates of 34 Mbps and an SNR of 3.24 are achieved, as shown in Fig. 5c, owing to the higher optical sensitivity of the Si-based APD to green wavelengths instead of UV wavelengths. The inset of Fig. 5c shows the corresponding eye diagram at 34 Mbps. In the case of CsPbBr_3_ perovskite NCs, the measured optical power illuminating the APD after the 500-nm LP filter is 0.508 µW. Given the similar optical powers illuminating the APD at two different wavelengths, i.e., 278 and 506 nm, a higher amplitude of the output voltage (*V*_out_), a higher received signal power, and a high SNR in the case of the CsPbBr_3_ perovskite layer can be expected on the basis of the following equation:

4 $$V_{\rm{out}} = P_{\rm{illuminance}} \times R_\lambda \times G$$

where*V*_out_ is the output voltage, *P*_illuminance_ is the power illuminating the APD, *R*_*λ*_ is the responsivity of the Si-based APD at different wavelengths, and *G* is the transimpedance gain. By using a colour-converting luminescent material at the receiver end, Dong et al. demonstrated enhanced signal and optical gain compared to a communication link directly using a blue LED^20^. However, in their work, low absorption was observed in the UV wavelength region, which restricted the application of their material to a UV-based communication link. We envisage that the achieved data rates can be further improved by using a more complex modulation scheme (e.g., orthogonal frequency-division multiplexing), pre-equalisation, bit loading and power allocation. Moreover, with the improvement in the modulation bandwidth of the solar-blind UVC LED and realisation of the UVC LD, a higher modulation bandwidth of up to hundreds of megahertz can be expected in the near future.

## Discussion

Table 1 summarises recently reported phosphor-based devices for optical wireless communication. Although the modulation bandwidth of the CsPbBr_3_ perovskite NC layer is lower than that in the prior work by Dursun et al.^2^, the PLQY in the present work is significantly higher by approximately 30% when the NCs are drop-cast in the form of a thin film, and thus, a higher photon conversion efficiency is exhibited that can improve photodetection. Moreover, the lower modulation bandwidth in the present work compared to prior work could be attributed to the competing band states and dynamics of recombination mechanisms in the CsPbBr_3_ perovskite NCs^45,46^. However, thorough investigations are still required to understand the mechanisms so that one can potentially manipulate the recombination dynamics favourable for the design of perovskite-based optoelectronic devices in the future. Compared to other prior works, our study highlighted the superior performance of the CsPbBr_3_ perovskite NC layer with a high-PLQY and a fast-PL decay time for a novel receiver design and potential monolithic integration with a Si-based receiver in a UV-based communication link.

**Table 1:** Comparison of phosphor-based devices for optical wireless communication

Material	PLQY	3-dB Bandwidth	Transmitter	Data Rate	Application module	Refs.
CsPbBr_3_	~70% (solution)	491 MHz	450 nm LD	2 Gbps	Transmitter	Dursun and Shen et al.^2^
BBEHP-PPV	≥75% (thin film)	≥200 MHz	450 nm LD	350 Mbps	Transmitter	Sajjad et al.^44^
RhB@Al-DBA	12% (thin film)	3.6 MHz	395 nm LED	3.6 Mbps	Transmitter	Wang et al.^47^
Carbon dots	21% (solution)	285 MHz	450 nm LD	350 Mbps	Transmitter	Zhou et al.^48^
CsPbBr_1.8_I_1.2_	78% (solution)	73 MHz	445 nm LED	190 Mbps	Transmitter	Mei et al.^49^
CdTe QD	69% (thin film)	42 MHz	–	–	Transmitter	Zhou et al.^50^
CPC-LSC	60% (thin film)	–	blue LED	400 Mbps	Receiver	Dong et al.^20^
CsPbBr_3_	72.95% (thin film)	70.92 MHz	278 nm LED	34 Mbps	Receiver (UVC)	The present work

In this study, a CsPbBr_3_ perovskite NC layer with a high PLQY (~73%) and a fast PL decay time (4.5 ns) was demonstrated as a colour-converting luminescent material for a novel UV photodetection scheme based on a low-cost and mature Si-based PD platform. Remarkably, nearly three-fold improvement in the responsivity and an approximately 25% increase in the EQE were observed. We also showed that it is possible to use the hybrid CsPbBr_3_-silicon colour convertor to achieve a large small-signal modulation bandwidth of 70.92 MHz and a high data rate of up to 34 Mbps in a solar-blind UVC communication link. Our approach based on a composition-tuneable perovskite-based phosphor exploits the feasibility of monolithic integration with low-cost and mature Si-based devices for high-speed UV photodetection. This study opens a new pathway for utilisation of perovskite-based material systems benefiting both terrestrial and underwater UV-internet systems.

## Materials and methods

### CsPbBr_3_ perovskite NCs preparation and characterisation

The CsPbBr_3_ QD solution was obtained from Quantum Solutions LLC (www.quantum-solutions.com). The QDs have oleic acid and oleylamine as ligands on the surface and are dispersed in toluene, with a QD concentration of approximately 20 mg/mL. TEM was performed on a Titan G2 80–300 (FEI Co.) operating at 300 kV. High-resolution TEM was carried out using an aberration-corrected and monochromated FEI Titan G2 80–300. Images were recorded on a charge coupled device (CCD) camera (2 k × 62 k, Gatan model 895) with a binning mode of two. Sample preparation for TEM was performed by diluting the QD solution using toluene, which was drop-cast onto a formvar/carbon-coated 300 mesh copper TEM grid for analysis. The CsPbBr_3_ NC layer was drop-cast onto a UV quartz substrate and dried to allow solvent evaporation for optical characterisation. With regard to UV–VIS absorption and PL characterisations, the steady-state reflection spectra were recorded using an Edinburg F900 spectrometer with an integrating sphere. Then, the Kubelka–Munk relation was used to convert the reflectance data into absorption spectra for CsPbBr_3_ NCs on the UV quartz substrate. The steady-state PL and time-resolved PL were recorded using an Edinburg F900 and a FluoroMax®-4 spectrometer, respectively. For time-resolved PL measurements, the instrument works on the principle of time-correlated single-photon counting (TCSPC); a 372-nm laser pulse was used as the excitation light source.

### Photoelectrical measurement

A 500-W Hg(Xe) arc lamp (Newport, 66142) was used as the light source and guided into a monochromator (Cornerstone^TM^, CS260) for wavelength tuning. The output light was then guided through a series of optical lenses and focused onto the input port of the integrating sphere (Newport, 819 series). The inner surface of the integrating sphere was coated with PTFE. A Si-based PIN junction photodiode (Thorlabs, FDS100) with the borosilicate window removed was mounted on the output port of the integrating sphere.

### Modulation bandwidth measurement

The setup consisted of a 375-nm LD (Thorlabs, L375P70MLD) mounted on a thermoelectric cooler (TEC). The output signal from a vector network analyser (Agilent, E5061B) was connected to the LD. The light was guided through UV plano-convex lenses (Thorlabs, LA1951A & Edmund Optics, No. 36–689) into the integrating sphere. The light re-emitted from CsPbBr_3_ NCs was filtered by a 500-nm LP filter (Thorlabs, FELH0500) and passed through two plano-convex lenses (Thorlabs, LA4148 and LA4052) and an objective lens (Thorlabs, LMU15X) before being collected by a Si-based APD (Thorlabs, APD430A2). The APD was connected to the vector network analyser. The vector network analyser was pre-calibrated with an E-calibration module (Agilent, 85093–60010) before the experiment.

### Data transmission measurement

A UVC LED (LG Innotek, LEUVA66H70HF00) was modulated through a bias tee (Mini-Circuits, ZFBT-4R2GW-FT+) and connected to a BERT transmitter (Anritsu, ME522A). Two plano-convex lenses (Thorlabs, LA4148 and LA4052) and an objective lens (Thorlabs, LMU15X) were used to guide the incoming light onto the Si-based APD. In the case of CsPbBr_3_ perovskite NCs, a 500-nm LP filter (Thorlabs, FELH0500) was added. For the UVC LED only, UV ND filters (Thorlabs, NDUV10A and NDUV06A) were added to reduce the light power and maintain it below the saturation power of the APD. The signal received from the APD was analysed by the BERT receiver (Anritsu, ME522A). The eye diagrams were simultaneously captured using a digital communication analyser (Agilent, 86100C Infiniium DCA-J Wideband Oscilloscope).

## Supplementary information

SUPPLEMENTARY INFORMATION for High-speed colour-converting photodetector with all-inorganic CsPbBr3 perovskite nanocrystals for ultraviolet light communication
