# Supplementary material for: High-speed colour-converting photodetector with all-inorganic CsPbBr3 perovskite nanocrystals for ultraviolet light communication
Source: Light Sci Appl. 2019 Oct 16;8:94. doi: 10.1038/s41377-019-0204-4 (PMC6804731; doi:10.1038/s41377-019-0204-4)
Supplement: Supplementary file 1 — SUPPLEMENTARY INFORMATION for High-speed colour-converting photodetector with all-inorganic CsPbBr3 perovskite nanocrystals for ultraviolet light communication [file 41377_2019_204_MOESM1_ESM.docx]

***Supplementary Information***

**High-speed colour-converting photodetector with all-inorganic CsPbBr_3_ perovskite nanocrystals for ultraviolet light communication**

Chun Hong Kang^1, §^, Ibrahim Dursun^2, §^, Guangyu Liu^1^, Lutfan Sinatra^3^, Xiaobin Sun^1^, Meiwei Kong^1^, Jun Pan^2^, Partha Maity^2^, Ee-Ning Ooi^1^, Tien Khee Ng^1^, Omar F. Mohammed^2^, Osman M. Bakr^2, *^, and Boon S. Ooi^1, *^

^1^Photonics Laboratory, Division of Computer, Electrical, and Mathematical Sciences and Engineering and ^2^Division of Physical Science and Engineering, King Abdullah University of Science and Technology (KAUST), Thuwal 23955-6900, Kingdom of Saudi Arabia

^3^Quantum Solutions LLC, Thuwal 23955-6900, Kingdom of Saudi Arabia

^§^C.H. Kang and I. Dursun contributed equally to this work.

*Corresponding authors: Osman M. Bakr (Email: osman.bakr@kaust.edu.sa, Tel. No: +966 (0)12 808-4582) and Boon S. Ooi (Email: boon.ooi@kaust.edu.sa, Tel. No: +966 (0)12 808-4350)

**
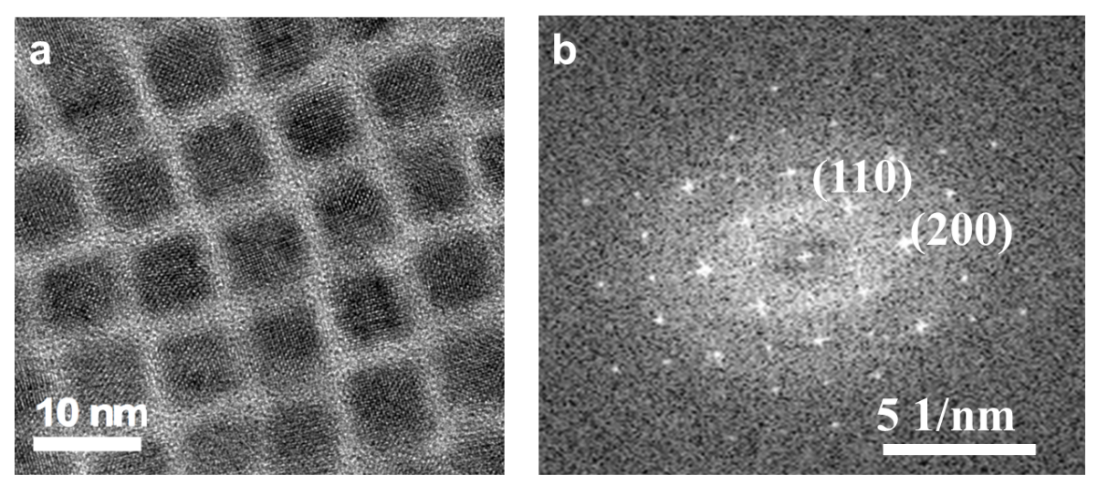
**

**Figure S1**. **a** High-resolution transmission electron microscopy (HR-TEM) image of CsPbBr_3_ perovskite nanocrystals. **b** Fast Fourier Transform (FFT) image from single nanocrystal.


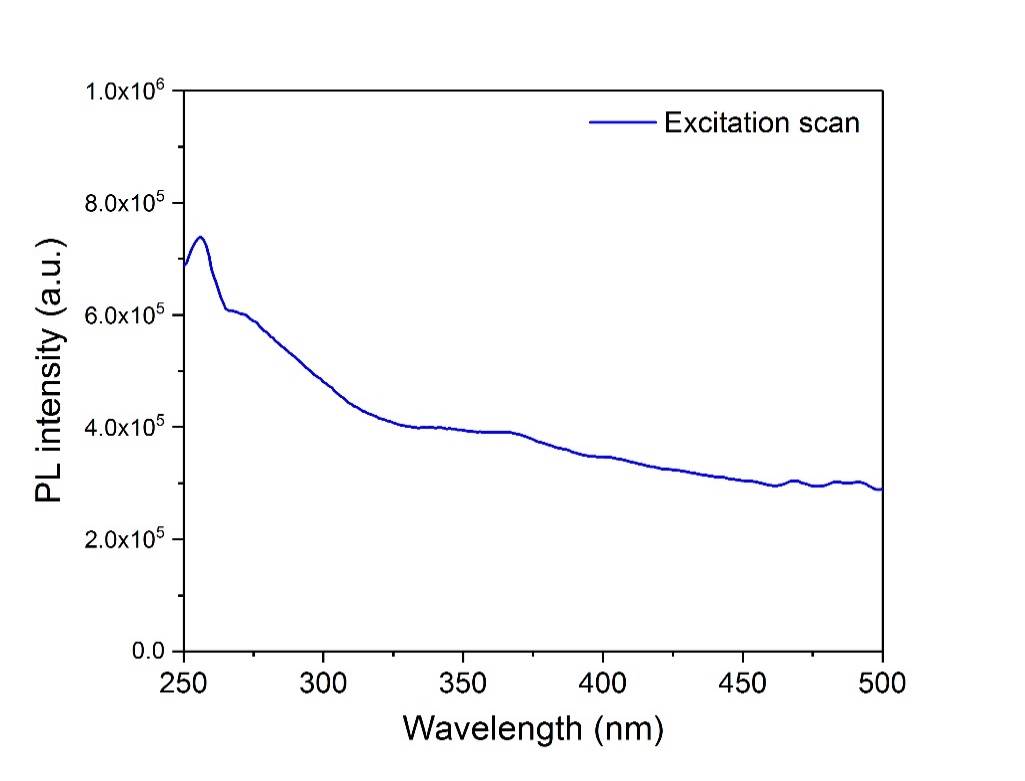


**Figure S2**. Excitation spectra of CsPbBr_3_ perovskite nanocrystals.


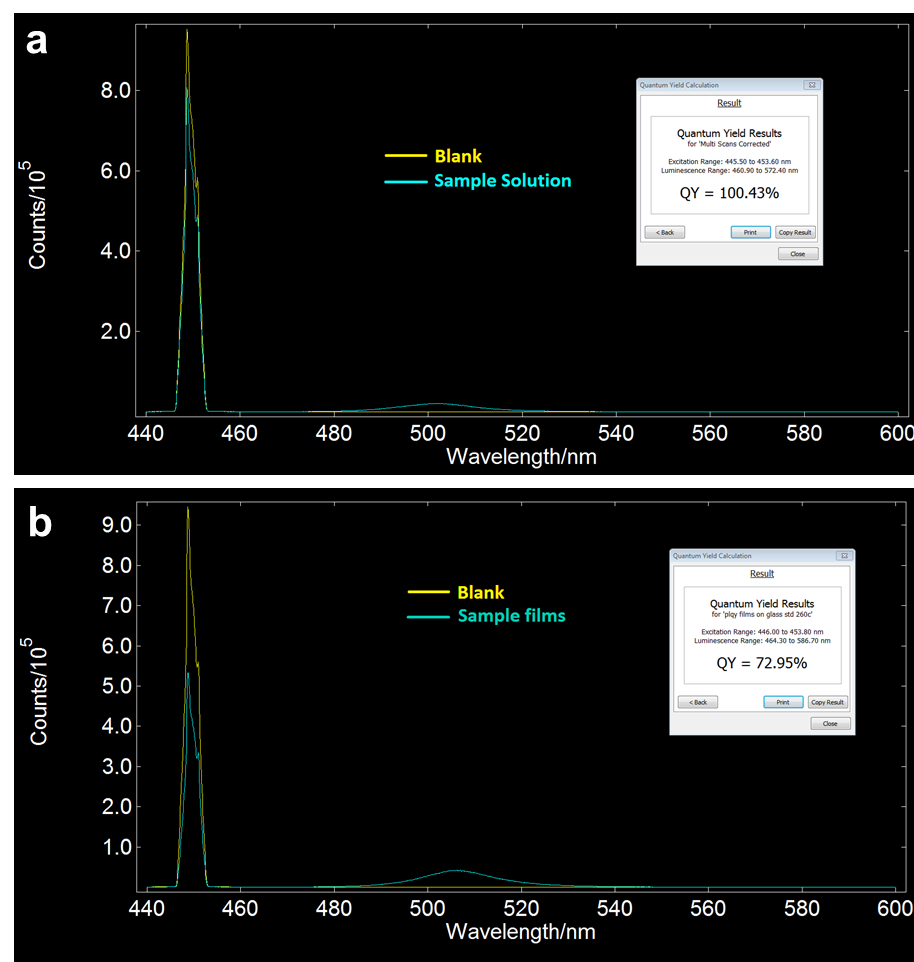


**Figure S3**. Photoluminescence quantum yield (PLQY) of CsPbBr_3_ perovskite NCs in **a** solution form and **b** drop-casted thin-film form on UV quartz substrate.


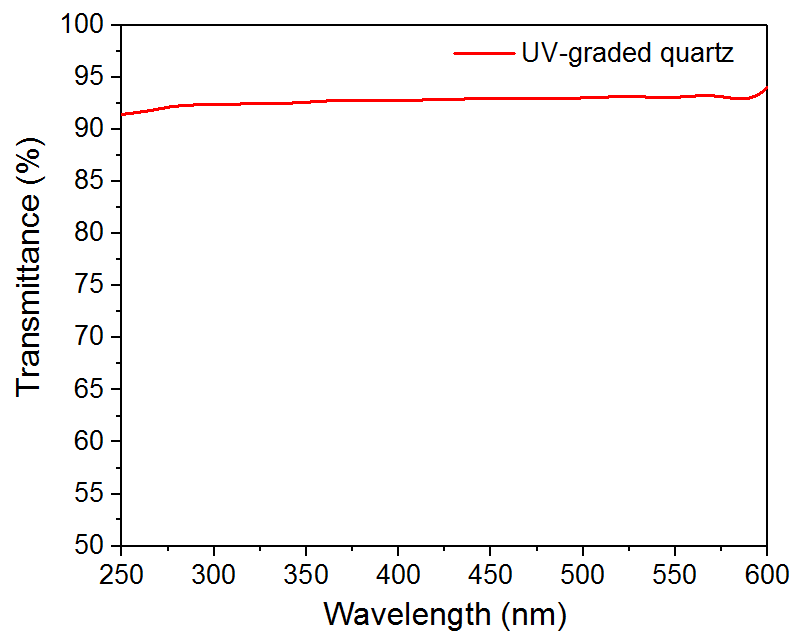


**Figure S4**. Transmittance spectrum of UV-graded quartz from UV to visible wavelength region.


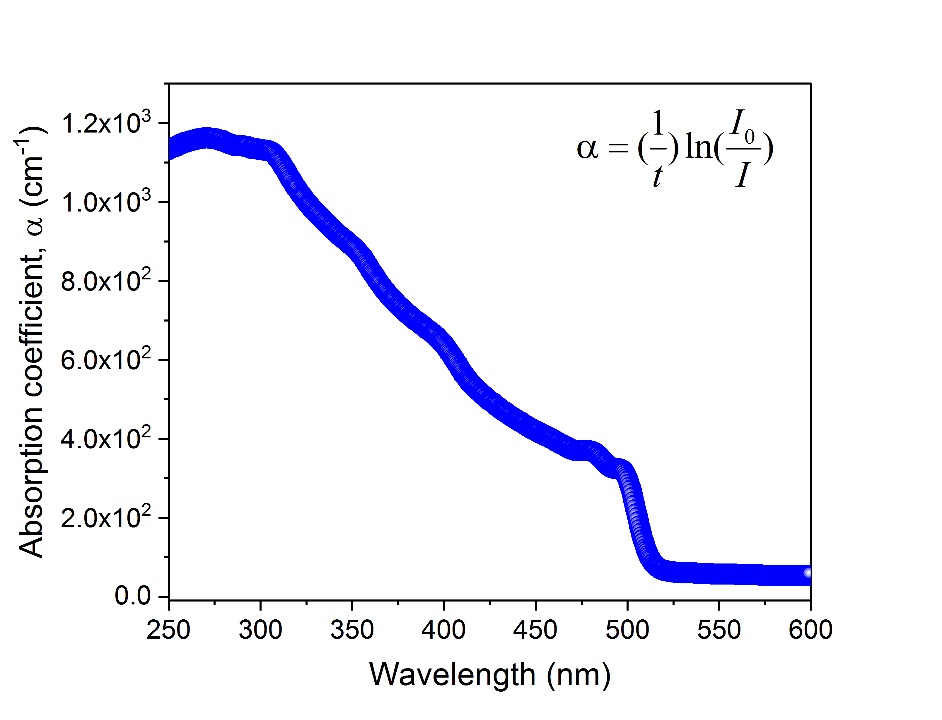


**Figure S5**. Absorption coefficient (α) of CsPbBr_3_ NCs films from 250 nm to 600 nm. The absorption coefficient was obtained from $\alpha=\left( \frac{1}{t} \right)\ln\left( \frac{I_{0}}{I} \right)=\left( \frac{1}{t} \right)ln(\frac{1}{T})$, where *t* represents layer thickness, *I_0_* represents incoming light intensity, *I* represents transmitted light intensity, and *T* represents transmittance.^1^

**
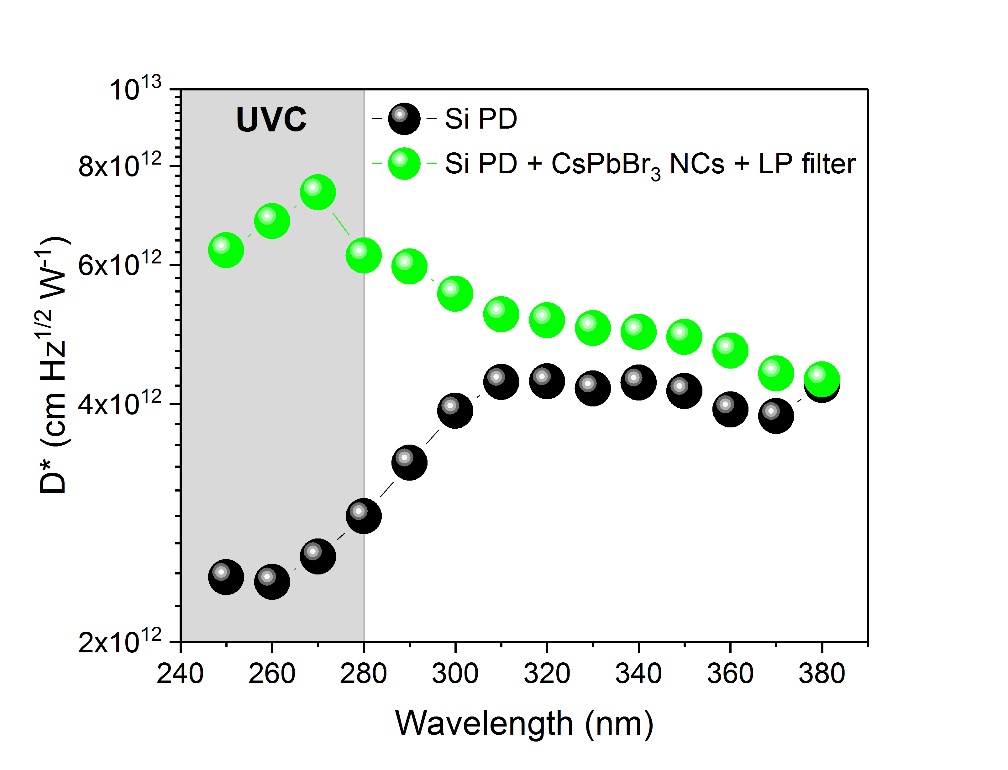
**

**Figure S6**. Specific detectivity (D*) of bare Si-based PD and hybrid CsPbBr_3_-Si photodetection scheme in the UV wavelength region.

**
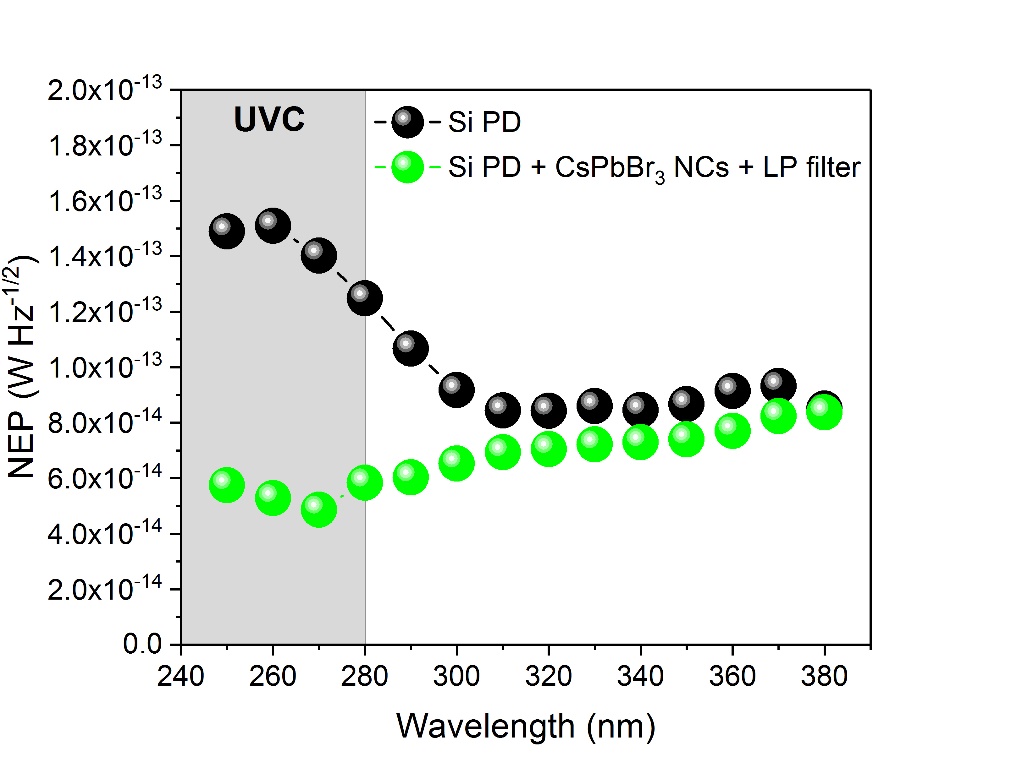
**

**Figure S7**. Noise equivalent power (NEP) of bare Si-based PD and hybrid CsPbBr_3_-Si photodetection scheme in the UV wavelength region.

**Table S1**. Performance of Si-based photodetector and hybridized Si-based photodetector with different material systems for improved performance.

| Material | Reverse Bias | Responsivity | EQE | Ref. |
| --- | --- | --- | --- | --- |
| Si | 5V | 0.03 A/W @ 250 nm  0.03 A/W @ 270 nm  0.04 A/W @ 300 nm | 13.5% @ 250 nm  13.3% @ 270 nm  18.3% @ 300 nm | FDS100 (Thorlabs) |
| Si | 10V | 0.034 A/W @ 250 nm  0.055 A/W @ 300 nm  0.054 A/W @ 350 nm | 16.9% @ 250 nm  22.7% @ 300 nm  19.1% @ 350 nm | FDS010 (Thorlabs) |
| Si | 5V | 0.030 A/W @ 350 nm  0.052 A/W @ 400 nm  0.12 A/W @ 450 nm | 10.6% @ 350 nm  16.1% @ 400 nm  33.1% @ 450 nm | FDS1010 (Thorlabs) |
| Si | 5V | 0.13 A/W @ 250 nm  0.10 A/W @ 275 nm  0.12 A/W @ 300 nm | 64.5% @ 250 nm  45.1% @ 275 nm  49.6% @ 300 nm | S1226-BQ (Hamamatsu) |
| Al_2_O_3_/Gr/Si | 0V | 0.14 A/W @ 250 nm  0.15 A/W @ 275 nm  0.16 A/W @ 300 nm | 69.44% @ 250 nm  67.64% @ 275 nm  66.13% @ 300 nm | Wan *et al*.^2^ |
| Nanoporous Si | 10V | 0.20 A/W @ 365 nm | 67.95% @ 365 nm | Ismail *et al.*^3^ |
| (F8)_9_BT:CBP/Si | 0V | 0.12 A/W @ 250 nm  0.15 A/W @ 300 nm  0.12 A/W @ 400 nm  0.21 A/W @ 500 nm | 59.5% @ 250 nm  62.0% @ 300 nm  37.2% @ 400 nm  52.1% @ 500 nm | Levell *et al.*^4^ |
| Gr/Porous Si | 1V | 0.19 A/W @ 400 nm | 58.9% @ 400 nm | Kim *et al.*^5^ |
| CsPbBr_3_/Si | 5V | 0.07 A/W @ 250 nm  0.08 A/W @ 270 nm | 35.06% (+ 22%) @ 250 nm  38.41% (+ 25%) @ 270 nm | **The present work** |


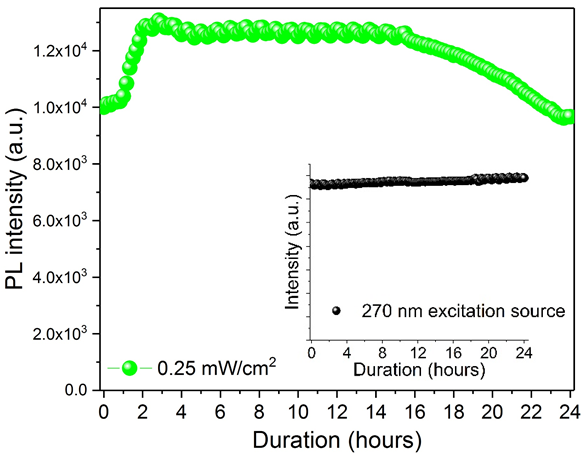


**Figure S8**. Photoluminescence (PL) intensity of CsPbBr_3_ perovskite NCs under continuous irradiation by 270-nm excitation source. The inset shows the light intensity of the excitation source.


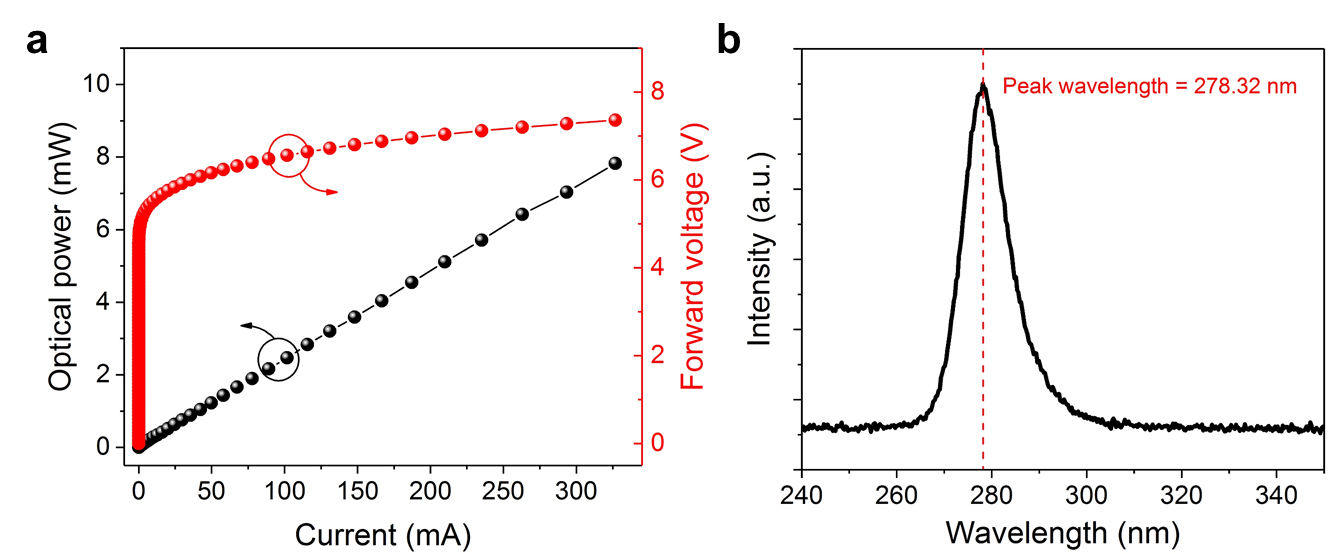


**Figure S9**. **a** *L-I-V* curves and **b** light emission spectrum of 278-nm UVC LED used in data rate measurement.


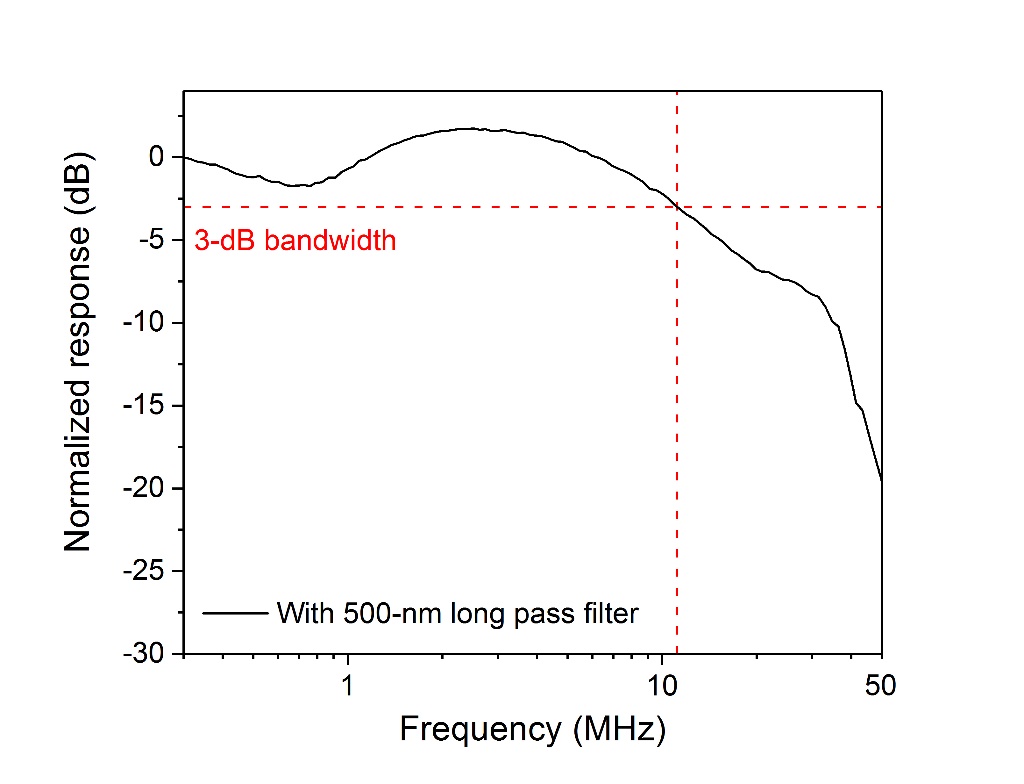


**Figure S10**. Normalized system response of 278-nm UVC LED, CsPbBr_3_ perovskite NCs with 500-nm long-pass filter and Si-based APD.

**REFERENCES**

1. Park, N.-G. Perovskite solar cells: an emerging photovoltaic technology. *Materials Today* **18,** 65-72 (2015).

2. Wan, X. *et al.* A self-powered high-performance graphene/silicon ultraviolet photodetector with ultra-shallow junction: breaking the limit of silicon? *npj 2D Materials and Applications* **1,** 4 (2017).

3. Ismail, R. A., Alwan, A. M. & Ahmed, A. S. Preparation and characteristics study of nano-porous silicon UV photodetector. *Applied Nanoscience* **7,** 9-15 (2017).

4. Levell, J. W., Giardini, M. E. & Samuel, I. D. W. A hybrid organic semiconductor/silicon photodiode for efficient ultraviolet photodetection. *Optics Express* **18,** 3219-3225 (2010).

5. Kim, J. *et al.* Near-ultraviolet-sensitive graphene/porous silicon photodetectors. *ACS Applied Materials & Interfaces* **6,** 20880-20886 (2014).
